# Towards Secretome Standardization: Identifying Key Ingredients of MSC-Derived Therapeutic Cocktail

**DOI:** 10.1155/2021/3086122

**Published:** 2021-08-26

**Authors:** Chiara Giannasi, Stefania Niada, Elena Della Morte, Sara Casati, Marica Orioli, Alice Gualerzi, Anna Teresa Brini

**Affiliations:** ^1^Department of Biomedical, Surgical and Dental Sciences, Università degli Studi di Milano, Milan, Italy; ^2^IRCCS Istituto Ortopedico Galeazzi, Laboratorio di Applicazioni Biotecnologiche, Milan, Italy; ^3^IRCCS Fondazione Don Carlo Gnocchi ONLUS, Milano, Italy

## Abstract

The therapeutic potential of the conditioned medium (CM) derived from MSCs (mesenchymal stem/stromal cells) in disparate medical fields, from immunology to orthopedics, has been widely suggested by *in vitro* and *in vivo* evidences. Prior to MSC-CM use in clinical applications, appropriate quality controls are needed in order to assess its reproducibility. Here, we evaluated different CM characteristics, including general features and precise protein and lipid concentrations, in 3 representative samples from adipose-derived MSCs (ASCs). In details, we first investigated the size and distribution of the contained extracellular vesicles (EVs), lipid bilayer-delimited particles whose pivotal role in intercellular communication has been extensively demonstrated. Then, we acquired Raman signatures, providing an overlook of ASC-CM composition in terms of proteins, lipids, and nucleic acids. At last, we analyzed a panel of 200 molecules including chemokines, cytokines, receptors, and inflammatory and growth factors and searched for 32 lipids involved in cell signalling and inflammation. All ASC-CM contained a homogeneous and relevant number of EVs (1.0 × 10^9^ ± 1.1 × 10^8^ particles per million donor ASCs) with a mean size of 190 ± 5.2 nm, suggesting the appropriateness of the method for EV retaining and concentration. Furthermore, also Raman spectra confirmed a high homogeneity among samples, allowing the visualization of specific peaks for nucleic acids, proteins, and lipids. An in depth investigation that focused on 200 proteins involved in relevant biological pathways revealed the presence in all specimens of 104 factors. Of these, 26 analytes presented a high degree of uniformity, suggesting that the samples were particularly homogenous for a quarter of the quantified molecules. At last, lipidomic analysis allowed the quantification of 7 lipids and indicated prostaglandin-E2 and N-stearoylethanolamide as the most homogenous factors. In this study, we assessed that ASC-CM samples obtained with a standardized protocol present stable features spanning from Raman fingerprint to specific marker concentrations. In conclusion, we identified key ingredients that may be involved in ASC-CM therapeutic action and whose consistent levels may represent a promising quality control in the pipeline of its preparation for clinical applications.

## 1. Introduction

Over the years, the transplantation of autologous or allogeneic stem cells, either naïve, differently primed, or genetically manipulated, has paved the way to the successful clinical management of several diseases whose pharmacological need was previously unmet. In particular, mesenchymal stem/stromal cells (MSCs), thanks to their regenerative and immunomodulatory potential [1, 2], have gained popularity as cell therapy in disparate clinical scenarios, from immunological diseases [3] to orthopedic conditions [4] and central nervous system injuries and disorders (e.g., traumatic brain injury, Parkinson's disease, and ischemic stroke) [5]. Besides the overall promising results, MSC transplantation (as well as cell-based therapy in general) entails evident drawbacks, such as ethical controversies, concerns linked to ex vivo expansion and high manufacturing costs. Starting from 2006 with the work of Gnecchi et al. [6], a growing body of evidence identifies paracrine signalling as the main effector of MSC therapeutic action, overturning the initial hypothesis that acknowledged cell engraftment, differentiation, and replacement as the main actors. Consequently, in 2010, Professor Caplan, the father of MSCs who firstly characterized and named them, proposed the new terminology of medicine signalling cells to highlight their secretory nature [7]. The term secretome, coined at the beginning of 2000's by Tjalsma and colleagues [8], defines the plethora of factors of different natures (lipids, nucleic acids, and proteins) secreted by a cell, both freely dissolved and conveyed into extracellular vesicles (EVs). The paradigm shift on MSC mode of action promoted cell secretome, intended both as an entire formula and as selected fractions (i.e., soluble and vesicular subcomponents), to a novel class of biological therapeutics. Indeed, the last few years witnessed the entrance of MSC secretome to several clinical trials, mostly in the regenerative medicine field, retracing the path of the clinical application of donor cells [9]. A critical search through ClinicalTrials.gov database, performed at the end of April 2021 using alternatively the keywords “secretome,” “conditioned medium,” or “extracellular vesicles” and the filter “interventional” as study type, lists a total of 14 studies based on MSC-secretome administration. Interestingly, most of these protocols relied on the use of CM (*n* = 11 versus *n* = 3 studies using EVs) derived from allogeneic MSCs (*n* = 8 versus *n* = 1 study specifically following an autologous setting). Thus far, only 3 of these studies are completed (NCT04315025, NCT03676400, and NCT04134676), but unluckily, there are no available results yet. Nevertheless, this picture allows us extrapolating some general considerations on the state of the art of MSC-based cell-free therapies:

- To date, the clinical use of complete secretome, accounting for both soluble and vesicular fractions, seems more easily applicable than isolated EVs. However, at the moment, EV potential in diagnostics remains pivotal, as confirmed by the high number of clinical trials relying on their use in this field
- Allogeneic settings are widely implemented, confirming the lack of immunogenicity and allowing to minimize interdonor variability and to avoid the need of performing additional procedures on patients for cell harvesting, thus excluding also donor site morbidity
- Donor MSCs are harvested from both neonatal (mostly umbilical cord) and adult (e.g., adipose tissue and bone marrow) tissue sources
- As for MSC-based cell therapy, the nature of the targeted pathologies is extremely various (among others, COVID-19 pneumonia, chronic wounds, alopecia, and osteoarthritis)

It is worth noting that up to now, the regulatory framework for the clinical use of cell secretome, or its subproducts, has not been clearly stated by any national nor international agency such as the FDA or the EMA. In the light of a successful translation to the clinics, there are still many technical issues to be addressed, mainly concerning the mode of action, scalability, standardization, and characterization.

In recent years, our research focused on the investigation of the conditioned medium (CM) from adipose tissue-derived MSCs (ASCs) in terms of biochemical composition [10–13] and therapeutic action, both *in vitro* [14, 15] and *in vivo* [16, 17]. Most of these studies provided the comparison between ASC-CM, consisting of both soluble factors and vesicle-conveyed ones, and ultracentrifuge-isolated EVs. Here, we decided to focus selectively on complete secretome. The present work takes a step forward in the perspective of ASC-CM characterization by quantifying a wide panel of molecules (cytokines, chemokines, receptors, growth and inflammatory factors, and bioactive lipids) in 3 different samples, in order to define some quality control criteria in the light of its future translation into clinics as an innovative cell-free therapeutic.

## 2. Materials and Methods

### 2.1. Cell Cultures

ASCs were isolated from the subcutaneous adipose tissue of 3 nonobese (BMI < 30) donors (1 male and 2 females, 54.7 ± 2.3 years old) who underwent total hip replacement surgery (*n* = 2) or liposuction (*n* = 1). All tissues were collected at IRCCS Istituto Ortopedico Galeazzi upon Institutional Review Board approval. Every donor provided a written informed consent. Adipose tissue samples were shredded with a sterile scalpel, digested for 30 min with 0.75 mg/ml type I collagenase (Worthington Biochemical Corporation, Lakewood, NJ, USA), and filtered with a 100 *μ*m cell strainer (Corning Incorporated, Corning, NY, USA). ASCs were grown in a culture medium composed by high-glucose DMEM (Sigma-Aldrich, St. Louis, MO, USA), 10% fetal bovine serum (FBS, Euroclone, Pero, Italy), 2 mM L-glutamine, 50 U/ml penicillin, and 50 *μ*g/ml streptomycin (Sigma-Aldrich, St. Louis, MO, USA) at 37°C, 5% CO_2_.

### 2.2. CM Production

ASCs from V to VII passage at 90% of confluence were incubated in starving conditions (without FBS) for 72 h. No signals of cell suffering were ever recorded during the period. The medium was then collected and centrifuged at 2500*g* for 15 min at 4°C with the purpose of eliminating cell debris, dead cells, and large apoptotic bodies. The supernatants were then concentrated about 60 times by centrifuging at 4000*g* for 90 min at 4°C in Amicon Ultra-15 Centrifugal Filter Devices with 3 kDa molecular weight cut-off (Merck Millipore, Burlington, MA, USA). This procedure allows the retention of the vesicular component of cell secretome, as previously demonstrated in [12, 13, 15]. The safety and efficacy of the final product obtained through this procedure have been already tested both *in vitro* [14, 15] and *in vivo* [16, 17].

### 2.3. Nanoparticle Tracking Analysis (NTA)

ASC-CM samples were appropriately diluted in 0.22 *μ*m triple-filtered PBS and analyzed by NanoSight NS3000 (Malvern Panalytical, Salisbury, UK). Three videos, each one lasting 1 min, have been recorded for every sample. All measurements respected the quality criteria of 20-120 particles/frame, concentration of 10^6^ − 4 × 10^9^ particles/ml, and number of valid tracks > 20%. After capture, the videos have been analyzed by the in-build NanoSight Software NTA.

### 2.4. Protein Array

Undiluted ASC-CM samples were analyzed by RayBiotech facility (RayBiotech, Norcross, GA, USA). The concentration (pg/ml) of 200 analytes of different natures (cytokines, chemokines, receptors, and inflammatory and growth factors) was investigated using the Quantibody® Human Cytokine Array 4000 Kit, a combination of Human Cytokine Array Q4, Human Chemokine Array Q1, Human Receptor Array Q1, Human Inflammation Array Q3, and Human Growth Factor Array Q1 (https://www.raybiotech.com/quantibody-human-cytokine-array-4000/). Obtained values were normalized on donor cell number (pg/10^6^ ASCs).

### 2.5. Raman Spectroscopy

ASC-CM samples were diluted in sterile saline solution and analyzed with the Raman microspectroscope (LabRAM Aramis, Horiba Jobin Yvon S.A.S., Lille, France) equipped with a 532 nm laser following a previously reported protocol [10, 13]. CM samples were deposited on a calcium fluoride slide and air-dried, and then, measurements were performed in the spectral ranges 600-1800 and 2600-3200 cm^−1^. At least 15 spectra per sample were acquired and processed (baseline correction, unit vector normalization, and postacquisition calibration) taking advantage of the integrated software LabSpec 6 (Horiba Jobin Yvon S.A.S., Lille, France).

### 2.6. Targeted UHPLC-MS/MS-Based Lipidomics

Polyunsaturated fatty acids, eicosanoids, endocannabinoids, and N-acylethanolamides were quantified on a QTRAP 5500 triple quadrupole linear ion trap mass spectrometer (Sciex, Darmstadt, Germany) coupled with an Agilent 1200 Infinity pump ultrahigh-pressure liquid chromatography (UHPLC) system (Agilent Technologies, Palo Alto, CA, USA) using the UHPLC-MS/MS methods previously reported [18]. Briefly, undiluted ASC-CM samples (approximately 200 *μ*l/sample) were spiked with deuterated internal standards and 1 ml of cold acetonitrile was added for protein precipitation. After centrifugation, the supernatants were extracted with a 4 ml of dichloromethane/isopropanol (8 : 2; *v*/*v*) and centrifuged again. The organic layer was separated, dried, and reconstituted in 60 *μ*l methanol. 3 *μ*l aliquot was analyzed for endocannabinoids and N-acylethanolamides. The remaining solution was added with 500 *μ*l hydrochloride acid (0.125 N) and 4 ml ethyl acetate/n-hexane (9 : 1; *v*/*v*). The organic phase was dried, and the residue was reconstituted in 60 *μ*l acetonitrile. 30 *μ*l aliquot of methanol obtained from the neutral extraction and 30 *μ*l aliquot from acid extraction were merged, and 10 *μ*l was analyzed for polyunsaturated fatty acids and eicosanoid determination. Data acquisition and processing were performed using Analyst®1.6.2 and MultiQuant®2.1.1 software (Sciex, Darmstadt, Germany), respectively.

### 2.7. Validation of Selected Proteins and Lipids

The validation of selected proteins was performed on 5 additional ASC-CM samples (deriving from cells harvested from 2 female and 3 male donors, mean age = 54.6 ± 22.3 years old). The Human Magnetic Luminex Screening Assay Rk4yTGNI (R&D Systems, Minneapolis, MN, USA) was customized to contain 5 molecules: MCP-4, PDGF-AA, TNF RI, DKK-1, and RAGE. Duplicates of each ASC-CM (50 *μ*l/sample) were tested, undiluted, and read through Bio-Plex Multiplex System (Bio-Rad, Milan, Italy) following standard procedures. Data analysis was performed with MAGPIX xPONENT 4.2 software (Luminex Corporation, Austin, TX, USA). The validation of SEA and PGE2 levels was performed through the UHPLC-MS/MS methods previously described on the CM derived from 5 additional ASC populations (all female donors, mean age = 49.0 ± 11.1 years old).

### 2.8. Data Analysis and Statistics

Statistics was performed with GraphPad Prism 7 software (GraphPad Software, La Jolla, CA, USA) and Excel. *p* values < 0.05 were considered statistically significant. NTA data were analyzed by the Kruskal-Wallis test to evaluate interdonor variability. For the Raman spectra, descriptive and multivariate statistical analyses were performed with Origin2021 (OriginLab, Northampton, MA, USA). Principal component analysis (PCA) was performed to reduce the dimensionality of Raman spectral datasets and to highlight differences between the spectra, with the resulting principal components (PC) representing these spectral differences with increasing percentage of variance. For protein array data, D'Agostino and Pearson omnibus normality test was used to determine whether the samples come from a Gaussian distribution. None of the datasets passed the normality test and correlation (Spearman *r*), and linear regression analyses were then performed accordingly. Coefficient of variation (CV, also known as relative standard deviation or RSD) was calculated as the ratio of the standard deviation to the mean. A CV < 33% was set as threshold. PCA and clustering were performed by ClustVis (https://biit.cs.ut.ee/clustvis). Process/pathway analysis was performed by STRING (https://string-db.org/) following default settings.

## 3. Results

**Figure 1:**
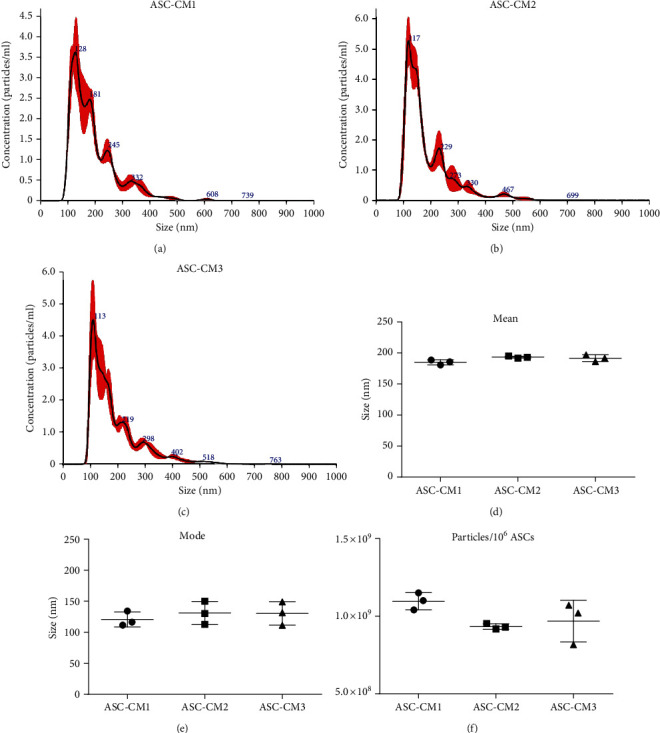
Dimensional characterization and quantification of ASC-CM extracellular vesicles. (a–c) Representative images of NTA referred to ASC-CM1 (a), ASC-CM2 (b), and ASC-CM3 (c). (d–f) Size distribution (d, e) and vesicular yield (f) deriving from 3 NTA measurements/sample. Data are shown as the mean ± SD.

CM samples were obtained, as previously described, from the culture medium harvested from confluent ASCs cultured for 3 days in serum-free conditions and concentrated by centrifugal filter devices of about 60 times. Since EVs represent a fundamental component of cell secretome, the first step of CM characterization focused on particle analysis. NTA revealed a similar size distribution between all the samples (Figures 1(a)–1(c)), with a mean EV size of 190 ± 5.2 nm (Figure 1(d)). Mode values (Figure 1(e)) further confirmed the homogeneity between preparations, indicating that the dimensions of the most frequently occurring particle populations ranged from 110 to 150 nm. All samples counted a relevant number of EVs, with an average of 1.0 × 10^9^ ± 1.1 × 10^8^ particles per million donor ASCs (Figure 1(f)), confirming the appropriateness of our protocol in retaining the vesicular component of cell secretome. No significant difference was observed in any parameter (nonparametric Kruskal-Wallis test, *p* > 0.05).

**Figure 2:**
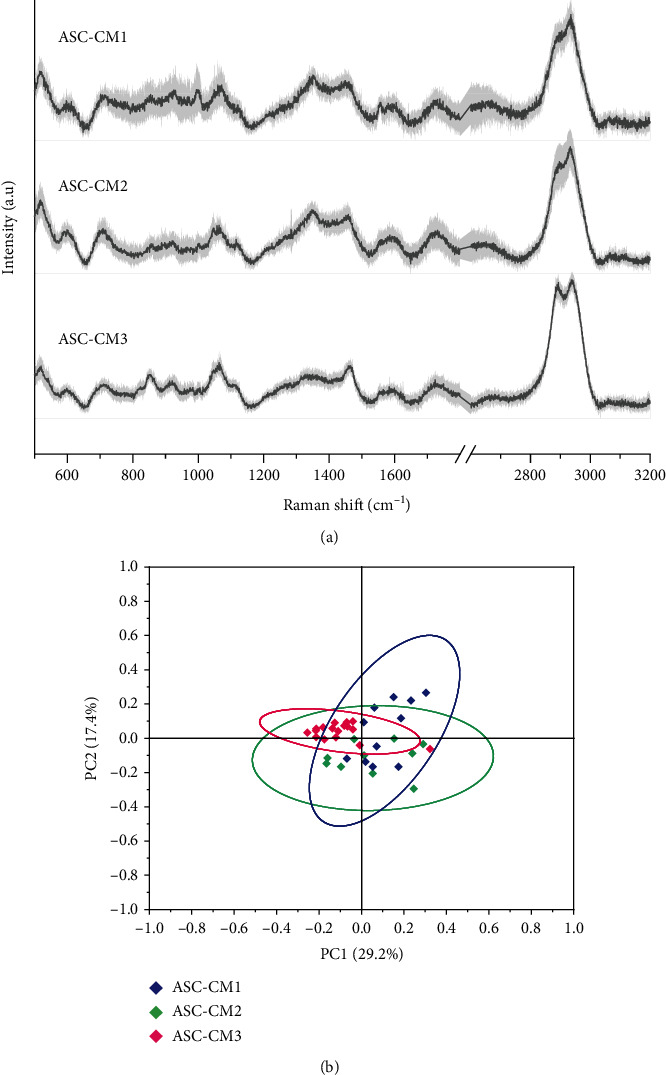
Raman spectroscopy analysis of ASC-CM samples. (a) Average Raman spectra obtained with 532 nm laser line on air-dried drops of ASC-CM samples. Gray shaded areas represent ±1 standard deviation. (b) Scatter plot of the PC1 and PC2 scores obtained for the 3 considered samples after multivariate statistical analysis (PCA). Ellipses represent the 95% confidence intervals calculated for each sample.

CM samples were then characterized by Raman spectroscopy, a vibrational spectroscopy method that was already proved to be effective in characterizing the soluble and the vesicular components of MSC secretome, verifying the purity and reproducibility of cell-free preparations [10, 13]. The obtained average spectra (Figure 2(a)) provide detailed biochemical information about the considered samples, with the Raman fingerprint accounting for proteins (amide I 1.650 cm^−1^), lipids (2700–3200 cm^−1^), and nucleic acids (720–820 cm^−1^), in agreement with previously reported data [13]. In particular, CM spectra showed a good signal-to-noise ratio and a good reproducibility, as assessed by the reported standard deviation (gray shaded areas in Figure 2(a)). The similarities in the chemical composition of the samples were further verified by multivariate statistical analysis: the PC1 and PC2 scores obtained for the three considered samples showed substantial overlap in the reported scatter plot (Figure 2(b)).

**Figure 3:**
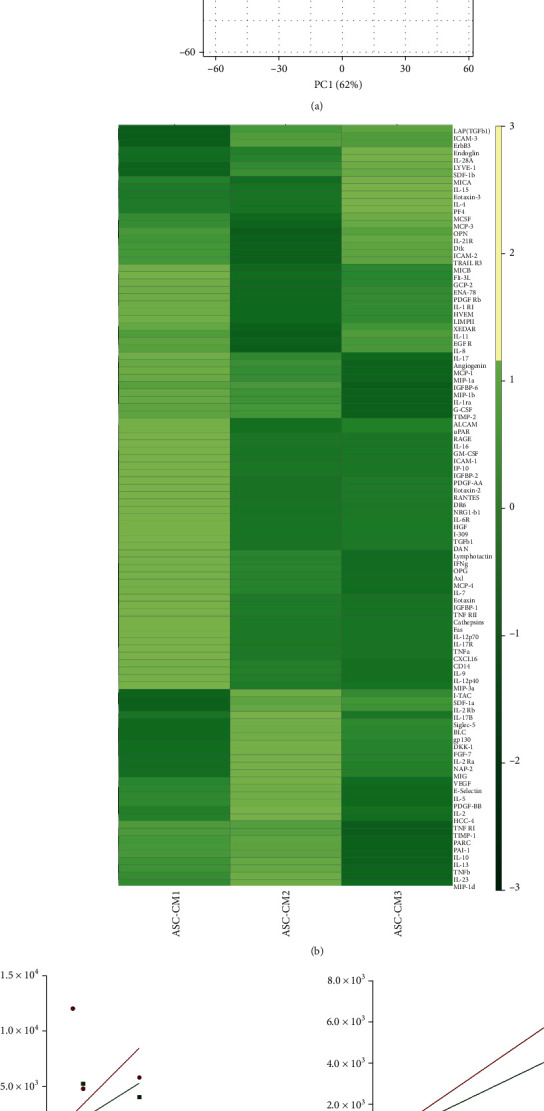
Clustering and correlation analysis of the 104 factors quantified in all ASC-CM samples. For the analyses, the levels of each analyte (pg/ml) were normalized on donor cell number and expressed as pg/10^6^ ASCs. (a) PCA plot and (b) heat map visualization of the protein levels in ASC-CM1, ASC-CM2, and ASC-CM3. (c, d) Correlation analysis of all the 104 factors (c) and of the 26 molecules (d) having a coefficient of variation below 33% (CV < 33%). For each graph, the equation of the regression lines is reported, together with Spearman *r* values.

**Figure 4:**
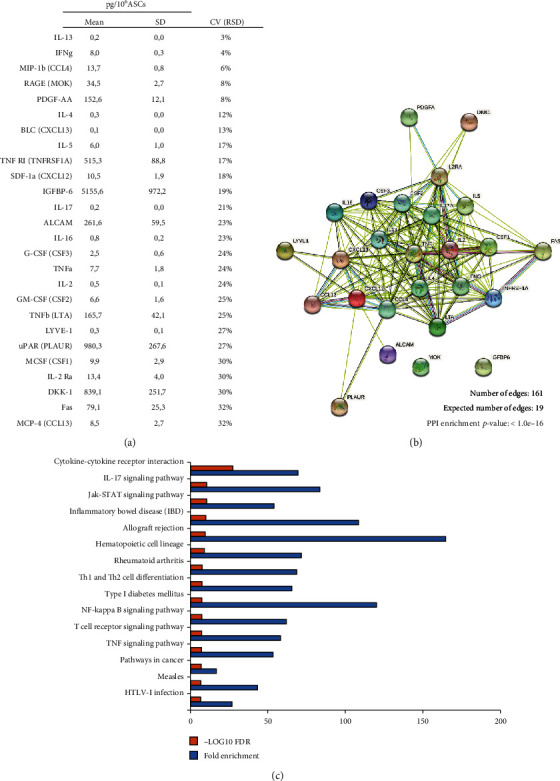
Protein interactions and functional prediction of the 26 most homogeneous factors quantified in ASC-CM samples. (a) List of the 26 selected factors having a coefficient of variation below 33% (CV < 33%) and (b) corresponding protein-protein interactions uncovered by STRING analysis. (c) Top 15 KEGG pathways associated with the 26 proteins selected based on false discovery rate (FDR) *p* value (-log_10_ FDR *p* values are reported as orange bars). Fold enrichment was calculated as follows: Fold enrichment = (observed protein count/number of most homogeneous factors)/(background gene count/total gene number) and is reported as blue bars.

In order to identify and quantify putative key factors involved in ASC-CM therapeutic action, we analyzed a panel of 200 chemokines, cytokines, receptors, and inflammatory and growth factors (40 molecules/category). 104 proteins were reliably quantified in all the samples (19 chemokines, 14 cytokines, 24 receptors, and 37 inflammatory and 10 growth factors), while 44 molecules were always undetectable (5 chemokines, 10 cytokines, 7 receptors, and 1 inflammatory and 21 growth factors) (Supplementary Tables 1 and 2). PCA on the 104 quantified factors unveiled a similar heterogeneity across the 3 samples (Figure 3(a)). The heat map further confirmed the lack of major differences among the specimens (Figure 3(b)). Correlation analysis, performed both on the entire datasets (Figure 3(c)) and on 26 selected factors with a coefficient of variation (CV) lower than 33% (Figure 3(d) and Figure 4(a)), showed a strong relationship between the quantitative variables among samples. Indeed, the slope of the regression lines always tended to 1 (Figures 3(c) and 3(d)). Moreover, Spearman *r* always resulted higher than 0.8, confirming a highly significant direct correlation between specimens (Figures 3(c) and 3(d), nonparametric Spearman correlation, *p* < 0.0001).

Since we aim at suggesting standards for CM quality control, further analyses focused on selected analytes particularly homogeneous across the samples. In details, around 25% (*n* = 26) of the quantified factors presented a CV < 33%, indicating a high degree of uniformity in all CM (Figure 4(a), Supplementary Table 3). Of note, 15 of these were inflammatory factors (Supplementary Table 3). STRING analysis underlined strict interconnections between these factors (Figure 4(b)). As expected, a strong enrichment in proteins involved in immune system regulation emerged by pathway analysis (Supplementary Table 4). In particular, the top 15 pathways ranked by FDR (Figure 4(c)) list proteins involved in cytokine-cytokine interaction (cytokine-cytokine receptor interaction/Jak-STAT signalling pathway) and T cell regulation (T cell receptor signalling pathway/Th1 and Th2 cell differentiation).

**Figure 5:**
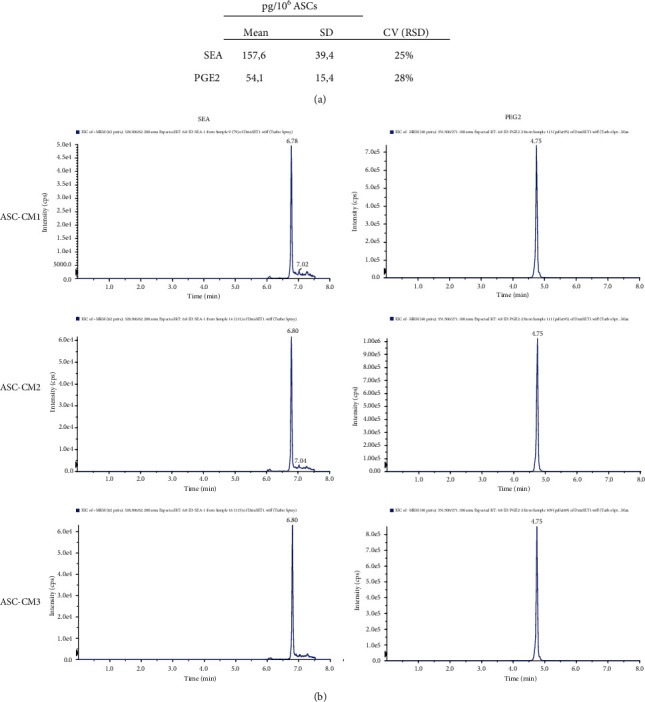
Lipid levels in ASC-CM samples. (a) Concentration of SEA and PGE2 in ASC-CM and (b) corresponding multiple reaction monitoring (MRM) chromatograms.

Besides proteins, lipids might also exert important roles in immune regulation. For this reason, in our CM samples, we analyzed a panel of endocannabinoids and eicosanoids known to be involved in inflammation. Seven lipid molecules, i.e., arachidonoyl acid (AA), eicosapentaenoyl acid (EPA), docosahexaenoic acid (DHA), prostaglandin-E2 (PGE2), prostaglandin-F2*α* (PGF2*α*), N-palmitoylethanolamide (PEA), and N-stearoylethanolamide (SEA) (Supplementary Table 5), were reliably quantified by UHPLC-MS/MS analysis in all ASC-CM samples. Except for 2-arachidonoylglycerol (2AG), quantified in 2 out of 3 samples, the other 24 lipids were always undetectable or unquantifiable (<LODs or LOQs). A coefficient of variation lower than 33% was found for SEA and PGE2 molecules (Figures 5(a) and 5(b), Supplementary Table 5), indicating a good degree of uniformity in the 3 CM. It is interesting to point out that, in CM, bioactive lipid by-products are more homogenous than precursors. This could suggest that mainly the firsts are released in a controlled fashion. Indeed, analyzing the pellets of the donor cells and also the precursors DHA, AA, and EPA presents strongly similar concentrations at intracellular level (Supplementary Table 6).

**Figure 6:**
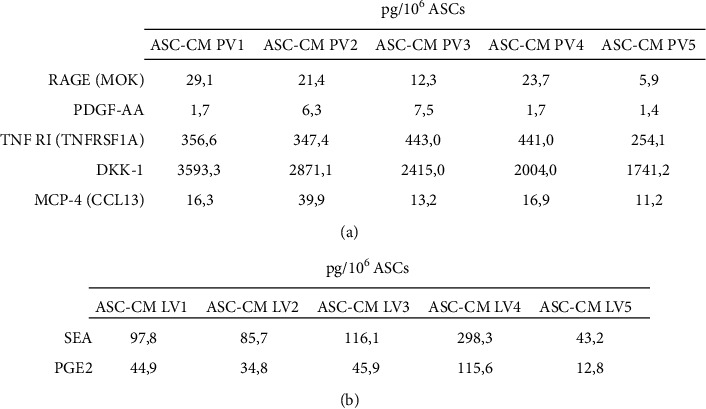
Validation of selected proteins and lipids. (a) Levels of 5 selected proteins (RAGE, PDGF-AA, TNF RI, DKK-1, and MCP-4) quantified in an ASC-CM protein validation (PV) cohort (*n* = 5). (b) SEA and PGE2 levels confirmed in an ASC-CM lipid validation (LV) cohort (*n* = 5). The levels of each analyte (pg/ml) were normalized on donor cell number and expressed as pg/10^6^ ASCs.

Since quantifying specific analytes could become a good quality control step for ASC-CM, we analyzed the concentration of a subset of factors in larger validation cohorts (*n* = 5 ASC-CM for both protein and lipid validation). Regarding proteins, our results confirmed both the presence and the homogeneity of the selected factors in all the analyzed samples (Figure 6(a)). Of note, while the mean concentrations of RAGE (18.5 ± 9.3 pg/10^6^ ASCs), TNF RI (368.4 ± 78.3 pg/10^6^ ASCs), and MCP-4 (19.5 ± 11.6 pg/10^6^ ASCs) nicely fit the ones observed in the original set (Figure 4(a)), the detected values for PDGF-AA (3.7 ± 2.9 pg/10^6^ ASCs) and DKK-1 (2524.9 ± 734.6 pg/10^6^ ASCs) are, respectively, lower and higher than expected. This discrepancy can be attributed to the implementation of distinct immunological techniques that therefore may have a different sensibility and specificity and may rely on the use of antibodies raised against disparate regions of the analytes. Conversely, lipid validation was performed through the same UHPLC-MS/MS methods used to test the original set. As shown in Figure 6(b), SEA (128.2 ± 98.8 pg/10^6^ ASCs) and PGE2 (50.8 ± 38.6 pg/10^6^ ASCs) were quantified in the entire ASC-CM lipid validation cohort within a concentration range that strongly overlaps what was previously observed (Figure 5(a)).

## 4. Discussion

The secretome from mesenchymal stem/stromal cells represents a mixture of biologically active ingredients whose individual role is still unknown. Nevertheless, their synergistic action in producing a clear therapeutic effect supports the rationale for investigating its clinical potential. This study is aimed at defining key elements of ASC secretome produced according to our protocol, which contemplates the culture of 90% confluent cells for 72 hours under serum deprivation and the following concentration of the conditioned medium through 3 kDa molecular weight cut off filters. Other groups currently implement a similar procedure [19, 20], although in literature, there are plenty of alternatives [21]. Therefore, according to the aphorism “the process is the product,” any change in the manufacturing process will undoubtedly affect the final product. Moreover, it should be pointed out that ASC-CM thus produced retains a substantial number of EVs and indeed previous evidences demonstrated a vesicular yield even higher than the one obtained by ultracentrifugation [13, 15].

**Figure 7:**
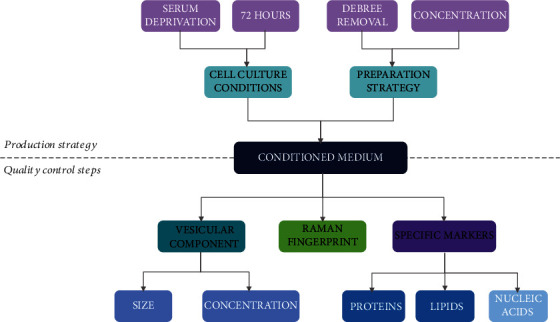
Scheme of our strategy for ASC-CM production together with the proposed quality control steps discussed in the text.

Here, we focused on key parameters that could be exploited either as general quality controls, such as vesicular component and Raman signature, or as specific markers, such as the quantification of selected proteins and lipids. A summary of the production process and the proposed quality controls is indicated in Figure 7. For the sake of completeness, even though nucleic acids such as miRNAs were not investigated in the current study, their role has been largely discussed by others (e.g., [22]) making them interesting candidates for additional or alternative quality control checks.

Given the biological relevance of EVs, their determination in CM was the first analysis performed in this study. EVs were abundant in all samples. Their number and size distribution were homogeneous and coherent with previous findings [13, 15]. Of note, the filtration protocol allows the retaining and concentration of the vesicular component with a process that is faster, easier, and less demanding than the gold-standard procedure (i.e., ultracentrifugation) [15]. Since EVs are strategic shuttles for biologicals, we suggest their quantification in CM preparations as a general quality control. Together with EV quantification, also Raman spectroscopy can provide a comprehensive picture of CM composition. It reveals the presence of macromolecules and points out differences and similarities across the samples, as reported here and in a previous study [13].

Differently, the investigation and quantification of selected factors could be adapted according to specific downstream applications.

The broad range analysis on 200 proteins playing pivotal roles in a variety of biological processes highlighted the presence of 26 highly conserved molecules in the 3 ASC-CM. Among these, we chose to validate 5 analytes, each belonging to a different panel: the chemokine MCP-4 (CV = 32%), the cytokine DKK-1 (CV = 30%), the receptor RAGE (CV = 8%), the inflammatory mediator TNF RI (CV = 8%), and the growth factor PDGF-AA (CV = 17%). For all these analytes, a high homogeneity among ASC-CM samples was confirmed. Given our promising *in vitro* [14, 15] and *in vivo* [17] results on the therapeutic action of ASC-CM in counteracting osteoarthritis (OA), herein, we focused our attention on the potential role of each molecule in this frame.

Monocyte chemoattractant protein 4 (MCP-4, also known as CCL13) is a member of the CC chemokine family that displays, besides a strong chemotaxis towards immune cells, a variety of immunomodulatory functions, spanning from induction of cytokine release to antimicrobial activity [23]. Interestingly, MCP-4 can undergo proteolytic cleavage by matrix metalloproteinases (MMPs), resulting in biologically active peptides that exert opposite actions on chemotaxis and inflammation [24]. This aspect is particularly intriguing since the aberrant MMP activity represents one of the milestones of OA progression [25]. In this perspective, ASC-CM therapeutic potential may rely also on the possibility of harnessing the anti-inflammatory properties of MCP-4 metabolites generated in situ by MMPs.

DKK-1 (Dickkopf-1) is a chondroprotective factor, acting as inhibitor of the Wnt/*β*-catenin signalling pathway. A massive activation of this pathway is involved in diseases like OA [26], where the conditional accumulation of *β*-catenin affects chondrocytes inducing a hypertrophic phenotype together with the overexpression of MMPs [27]. Interestingly, recent *in vitro* evidences described positive changes in the expression of *β*-catenin by subchondral osteoblasts following the administration of DKK-1 [28]. Consequently, its abundance in ASC-CM may represent a promising cue in counteracting OA progression.

The receptor for advanced glycation end products (RAGE) appeared remarkably homogenous in ASC-CM samples. Physiologically and pathologically, this is a transmembrane receptor whose activation by ligand interaction triggers intracellular signalling leading to increased release of reactive oxygen species and proinflammatory cytokines [29]. The presence of RAGE in ASC-CM cannot induce such a response so it might act as a decoy receptor. This could be exploited for the treatment of pathologies presenting a reduction of soluble RAGE (sRAGE, the “conventional” RAGE decoy receptor), together with an increase of its ligands. In OA, a reduction of sRAGE is associated with an increase in AGE levels in the synovial fluid [29, 30]. In this context, we hypothesize that ASC-CM injection in a limited space such as the synovial environment could mitigate the pathological sRAGE/AGE unbalance.

A similar consideration can fit for TFN RI (TNFRSF1A). This receptor is usually involved in the transduction of various inflammatory/stress stimuli by the activation of NF-*κ*B and the consequent transcription of specific genes leading to the production of proinflammatory and catabolic factors [31]. Again, the molecule present in ASC-CM medium cannot trigger these intracellular events while it could compete with cellular receptor. This could be of particular relevance in the treatment of pathologies associated with a relevant increase in TNF, such as rheumatoid arthritis [32], Crohn's disease [33], and OA [34]. In the latter, ASC-CM intra-articular administration could be particularly beneficial since the increase in TNF in the synovial fluid, synovial membrane, cartilage, and subchondral bone is also associated with an increased TNFRI in synovial fibroblasts, further amplifying the noxious signalling [34].

Platelet-derived growth factors (PDGFs) are key players in bone metabolism, and, in particular, PDGF-AA is involved in the crosstalk between subchondral bone and articular cartilage during OA onset [35]. Moreover, recent evidences suggest that PDGF-AA promotes remyelination and increases tissue repair in a rat model of spinal cord injury, overall improving the locomotor functional recovery [36].

Since recently, the involvement of lipids in physiological and pathological processes has been widely demonstrated; in our opinion, their analysis holds paramount importance. With the advent of the next-generation mass spectrometry (MS), significant advances occurred in the field of lipidomics. Our UHPLC-MS/MS method [18] is aimed at profiling a high number of bioactive lipids belonging to structurally similar classes, including polyunsaturated fatty acids, eicosanoids, endocannabinoids, and N-acylethanolamides. Since it is conceivable that these lipids released by ASCs may play a role in inflammatory processes, we performed an absolute quantification of 32 molecules thanks to the high sensitivity and specificity of triple quadrupole mass spectrometry and the use of labeled lipids. Among quantified lipids, SEA and PGE2 showed a relevant uniformity that was therefore validated in a larger ASC-CM cohort. SEA is an endogenous lipid belonging to the N-acylethanolamides family that acts as an anti-inflammatory/immunomodulatory agent through the downregulation of several proinflammatory cytokines [37]. Conversely, PGE2 exerts a well-known inflammatory action. Nevertheless, in the OA context, it can exert either anabolic or catabolic effects on chondrocytes and synoviocytes depending on its concentration [38–40]. Evidence suggests also that PGE2 may have an immune stimulatory role by facilitating Th1 differentiation and expanding Th17 T cells [37].

## 5. Conclusions

In conclusion, in this work, we identified key ingredients of ASC secretome that may be involved in its therapeutic action and whose stable levels among different ASC-CM batches may represent promising quality control criteria. Indeed, these indications may be relevant for a rapid and convenient reproducibility assessment of ASC-CM prior its use for different applications.

Nevertheless, we suggest not to focus reservedly on selected components but rather to aim at acquiring an overview of the great complexity of this promising cell-free therapeutic, whose strength relies precisely on the presence of a multitude of biologically active factors of different natures. Here, we propose multiple steps for secretome standardization, either providing an overlook of its composition by NTA and Raman spectroscopy or specifically focusing on the quantification of key molecules of different natures.

## Data Availability

All data used to support the findings of this study are included within the supplementary information files and/or are uploaded in zenodo repository (https://doi.org/10.5281/zenodo.5211269) and/or are available from the corresponding author upon request.

## Supplementary Materials

Supplementary Materials Supplementary Table 1: protein concentration (pg/ml) of factors quantified in all ASC-CM samples. Supplementary Table 2: list of factors undetectable in all ASC-CM samples. Supplementary Table 3: factors quantified in all ASC-CM samples, normalized on donor cell number, and ranked by coefficient of variation (CV). Supplementary Table 4: KEGG pathway enrichment analysis by STRING (FDR < 0.05). Supplementary Table 5: lipids quantified in all ASC-CM samples, normalized on donor cell number, and ranked by coefficient of variation (CV). Supplementary Table 6: lipids quantified in all ASC pellets, normalized on donor cell number, and ranked by coefficient of variation (CV).
