# Supplementary material for: Towards Secretome Standardization: Identifying Key Ingredients of MSC-Derived Therapeutic Cocktail
Source: Stem Cells Int. 2021 Aug 26;2021:3086122. doi: 10.1155/2021/3086122 (PMC8413055; doi:10.1155/2021/3086122)
Supplement: Supplementary Materials — Supplementary Table 1: protein concentration (pg/ml) of factors quantified in all ASC-CM samples. Supplementary Table 2: list of factors undetectable in all ASC-CM samples. Supplementary Table 3: factors quantified in all ASC-CM samples, normalized on donor cell number, and ranked by coefficient of variation (CV). Supplementary Table 4: KEGG pathway enrichment analysis by STRING (FDR < 0.05). Supplementary Table 5: lipids quantified in all ASC-CM samples, normalized on donor cell number, and ranked by coefficient of variation (CV). Supplementary Table 6: lipids quantified in all ASC pellets, normalized on donor cell number, and ranked by coefficient of variation (CV). [file 3086122.f1.zip › 3086122.f1.pdf]

Supplementary table 1_Factors quantified in all ASC-CM samples

	[] pg/ml			
Protein ID	Mean	SD		Chemokines
OPN	28,316.2	17,490.5		Cytokines
PF4	3,092.0	4,143.4		Receptors
Lymphotactin	2,079.9	255.4		Inflammatory factors
IL-9	1,723.1	1,237.5		Growth factors
CXCL16	1,105.5	887.1		
ENA-78	1,043.0	886.2		
Axl	577.7	108.6		
GCP-2	390.7	327.1		
Eotaxin-3	207.1	326.7		
MCP-3	162.2	177.2		
I-TAC	101.5	49.4		
SDF-1a	71.3	28.8		
MCP-4	53.5	6.0		
IP-10	46.9	43.1		
IL-28A	36.0	45.4		
MIP-3a	5.3	2.5		
HCC-4	2.9	2.7		
NAP-2	1.1	0.7		
PARC	0.5	0.2		
DKK-1	5,608.8	2,145.4		
gp130	4,224.3	2,181.2		
PAI-1	2,728.5	565.9		
IL-17B	2,294.7	895.3		
Cathepsin S	1,722.1	597.3		
DAN	1,639.0	2,009.9		
Angiogenin	589.2	52.5		
ICAM-2	567.7	414.5		
LAP(TGFb1)	417.0	364.1		
IL-23	387.1	292.3		
IL-2 Rb	265.4	192.6		
SDF-1b	129.3	95.7		
IL-2 Ra	89.7	33.8		
Siglec-5	65.4	44.4		
uPAR	6,346.8	1,647.3		
CD14	2,689.0	521.9		
ALCAM	1,701.7	427.9		
HVEM	860.4	428.6		
PDGF Rb	858.2	426.5		
Fas	501.7	88.9		
XEDAR	264.6	121.6		
Endoglin	255.4	237.9		
RAGE	228.0	60.8		
LIMPII	221.7	144.6		
TRAIL R3	174.6	129.0		
DR6	152.3	79.9		
MICB	149.7	55.7		
IL-21R	118.0	86.4		
MICA	83.1	67.9		
Flt-3L	66.6	27.7		
ICAM-3	39.1	25.1		
IL-17R	32.1	50.7		
Dtk	23.0	22.6		
E-Selectin	7.2	3.0		
NRG1-b1	6.1	1.6		
ErbB3	4.2	3.0		
LYVE-1	2.1	1.2		

IL-1 RI	1.3	1.1
TIMP-2	3,762.6	464.3
TNF RI	3,324.5	479.0
TIMP-1	1,775.5	310.6
ICAM-1	1,298.0	463.6
TNFb	1,058.3	178.7
RANTES	517.5	426.9
TNF RII	324.0	210.5
MCP-1	300.5	41.1
IL-8	137.6	87.2
IL-6R	122.3	61.9
IL-1ra	109.0	32.6
MIP-1b	90.5	23.1
MCSF	69.0	40.5
Eotaxin	66.7	14.3
IFNg	52.8	15.0
TNFa	49.4	9.3
PDGF-BB	44.4	35.0
GM-CSF	42.7	9.0
IL-5	39.4	8.6
IL-11	26.2	21.4
IL-7	24.0	9.2
G-CSF	15.9	0.9
MIP-1a	13.0	4.7
MIG	10.8	10.0
I-309	5.2	1.6
IL-16	5.1	1.1
IL-12p40	4.2	0.8
IL-2	3.3	0.9
IL-15	3.2	2.3
IL-4	2.2	1.0
Eotaxin-2	1.7	0.5
IL-13	1.4	0.4
IL-17	1.3	0.1
IL-10	0.8	0.3
BLC	0.7	0.2
IL-12p70	0.6	0.3
MIP-1d	0.6	0.4
IGFBP-6	33,092.5	3,758.1
TGFb1	30,732.6	26,258.4
HGF	2,902.6	773.3
IGFBP-2	1,721.6	2,007.6
PDGF-AA	1,009.7	279.3
EGF R	955.5	532.4
VEGF	642.9	247.5
OPG	443.6	65.3
FGF-7	365.2	244.0
IGFBP-1	68.3	26.4
