# Supplementary material for: Towards Secretome Standardization: Identifying Key Ingredients of MSC-Derived Therapeutic Cocktail
Source: Stem Cells Int. 2021 Aug 26;2021:3086122. doi: 10.1155/2021/3086122 (PMC8413055; doi:10.1155/2021/3086122)
Supplement: Supplementary Materials — Supplementary Table 1: protein concentration (pg/ml) of factors quantified in all ASC-CM samples. Supplementary Table 2: list of factors undetectable in all ASC-CM samples. Supplementary Table 3: factors quantified in all ASC-CM samples, normalized on donor cell number, and ranked by coefficient of variation (CV). Supplementary Table 4: KEGG pathway enrichment analysis by STRING (FDR < 0.05). Supplementary Table 5: lipids quantified in all ASC-CM samples, normalized on donor cell number, and ranked by coefficient of variation (CV). Supplementary Table 6: lipids quantified in all ASC pellets, normalized on donor cell number, and ranked by coefficient of variation (CV). [file 3086122.f1.zip › 3086122.f2.pdf]

Supplementary table 2_Factors undetectable in all ASC-CM samples

Protein ID	
6Ckine	Chemokines
IL-17F	Cytokines
TARC	Receptors
TECK	Inflammatory factors
TSLP	Growth factors
Cripto-1	
E-Cadherin	
EpCAM	
FAS L	
Fcg RIIBC	
IL-13 R1	
IL-13 R2	
NrCAM	
IL-1 R4	
TRAIL R4	
B7-1	
BCMA	
CD30	
CEACAM-1	
IL-2 Rg	
PECAM-1	
TIM-1	
IL-1a	
AR	
BMP-7	
b-NGF	
EGF	
EG-VEGF	
FGF-4	
GDNF	
GH	
HB-EGF	
IGF-1	
Insulin	
MCSF R	
NGF R	
NT-4	
SCF	
SCF R	
TGFa	
TGFb3	
VEGF R2	
VEGF R3	
VEGF-D	
