# Supplementary material for: Towards Secretome Standardization: Identifying Key Ingredients of MSC-Derived Therapeutic Cocktail
Source: Stem Cells Int. 2021 Aug 26;2021:3086122. doi: 10.1155/2021/3086122 (PMC8413055; doi:10.1155/2021/3086122)
Supplement: Supplementary Materials — Supplementary Table 1: protein concentration (pg/ml) of factors quantified in all ASC-CM samples. Supplementary Table 2: list of factors undetectable in all ASC-CM samples. Supplementary Table 3: factors quantified in all ASC-CM samples, normalized on donor cell number, and ranked by coefficient of variation (CV). Supplementary Table 4: KEGG pathway enrichment analysis by STRING (FDR < 0.05). Supplementary Table 5: lipids quantified in all ASC-CM samples, normalized on donor cell number, and ranked by coefficient of variation (CV). Supplementary Table 6: lipids quantified in all ASC pellets, normalized on donor cell number, and ranked by coefficient of variation (CV). [file 3086122.f1.zip › 3086122.f3.pdf]

Supplementary table 3_Factors quantified in all ASC-CM samples, normalized on donor cell number and ranked by CV

Protein ID	Mean	SD	CV (RSD)
	pg/10^6 ASC		
IL-13	0.21	0.01	3%
IFNg	7.95	0.28	4%
MIP-1b (CCL4)	13.70	0.76	6%
RAGE (MOK)	34.51	2.68	8%
PDGF-AA (PDGFA)	152.64	12.09	8%
IL-4	0.33	0.04	12%
BLC (CXCL13)	0.10	0.01	13%
IL-5	6.03	1.00	17%
TNF RI (TNFRSF1A)	515.32	88.78	17%
SDF-1a (CXCL12)	10.48	1.85	18%
IGFBP-6 (IGFBP-6)	5155.56	972.21	19%
IL-17	0.20	0.04	21%
ALCAM	261.62	59.48	23%
IL-16	0.80	0.18	23%
G-CSF (CSF3)	2.50	0.59	24%
TNFa	7.67	1.82	24%
IL-2	0.51	0.12	24%
GM-CSF (CSF2)	6.62	1.63	25%
TNFb (LTA)	165.67	42.07	25%
LYVE-1	0.30	0.08	27%
uPAR (PLAUR)	980.33	267.55	27%
MCSF (CSF1)	9.89	2.92	30%
IL-2 Ra (CD25; ISG20)	13.45	4.03	30%
DKK-1	839.06	251.72	30%
Fas	79.08	25.31	32%
MCP-4 (CCL13)	8.51	2.73	32%
XEDAR	39.64	13.63	34%
Angiogenin	95.31	34.34	36%
TIMP-1	286.45	103.41	36%
PAI-1	440.21	164.68	37%
TIMP-2	611.26	228.99	37%
Lymphotactin	334.90	125.55	37%
I-TAC	14.87	5.73	39%
NRG1-b1	0.97	0.38	40%
OPN	4097.67	1636.69	40%
MICB	23.11	9.24	40%
IL-15	0.44	0.18	40%
OPG	71.72	28.74	40%
MCP-1	49.04	19.83	40%
IL-17B	351.01	142.07	40%
HGF	460.49	193.70	42%
Eotaxin	10.71	4.53	42%
CD14	435.13	188.45	43%
EGF R	141.73	61.53	43%
IL-12p40	0.67	0.29	44%
Axl	93.98	41.46	44%
ICAM-3	5.49	2.49	45%
VEGF	102.23	48.00	47%
Eotaxin-2	0.27	0.13	48%
IL-21R	16.71	8.17	49%
SDF-1b	17.68	8.72	49%
gp130	626.00	309.55	49%
Flt-3L	10.39	5.15	50%
IL-10	0.13	0.06	50%
I-309	0.83	0.42	50%
ICAM-2	80.71	40.52	50%
IL-8	20.24	10.25	51%
E-Selectin	1.16	0.59	51%
MICA	11.35	5.77	51%
TRAIL R3	24.80	12.71	51%
IL-1ra	18.30	9.48	52%
PDGF Rb	131.76	68.27	52%
HVEM	132.68	71.38	54%
PARC	0.08	0.04	54%
ErbB3	0.59	0.33	56%
ICAM-1	211.12	120.47	57%
MIP-1a	2.21	1.28	58%
Cathepsin S	283.54	165.78	58%
IL-7	4.06	2.57	63%
IGFBP-1	11.38	7.21	63%
Endoglin	33.61	21.96	65%
NAP-2	0.17	0.11	67%
IL-11	3.78	2.59	69%
IL-12p70	0.10	0.07	69%
IL-2 Rb	37.44	26.36	70%
MIP-3a	0.91	0.65	71%
Siglec-5	9.58	6.89	72%
FGF-7	54.53	39.58	73%
IL-6R	20.26	14.81	73%
LAP(TGFb1)	55.56	40.97	74%

Chemokines
Cytokines
Receptors
Inflammatory factors
Growth factors

DR6	25.43	19.23	76%
LIMPII	34.76	26.40	76%
IL-23	66.22	51.74	78%
MIP-1d	0.10	0.08	78%
Dtk	3.18	2.50	79%
PDGF-BB	7.51	6.12	81%
MCP-3	21.68	18.94	87%
TNF RII	56.52	49.60	88%
IL-1 RI	0.21	0.19	91%
IL-9	307.09	282.27	92%
HCC-4	0.48	0.47	97%
ENA-78	163.63	158.88	97%
CXCL16	196.07	199.24	102%
GCP-2	63.64	64.96	102%
MIG	1.64	1.72	105%
RANTES	89.38	93.87	105%
TGFb1	5370.64	5781.30	108%
IL-28A	4.38	4.79	109%
IP-10	8.36	9.42	113%
PF4	379.29	429.50	113%
IGFBP-2	317.66	423.03	133%
DAN	301.42	420.59	140%
Eotaxin-3	23.95	35.29	147%
IL-17R	6.36	10.19	160%
