# Supplementary material for: Towards Secretome Standardization: Identifying Key Ingredients of MSC-Derived Therapeutic Cocktail
Source: Stem Cells Int. 2021 Aug 26;2021:3086122. doi: 10.1155/2021/3086122 (PMC8413055; doi:10.1155/2021/3086122)
Supplement: Supplementary Materials — Supplementary Table 1: protein concentration (pg/ml) of factors quantified in all ASC-CM samples. Supplementary Table 2: list of factors undetectable in all ASC-CM samples. Supplementary Table 3: factors quantified in all ASC-CM samples, normalized on donor cell number, and ranked by coefficient of variation (CV). Supplementary Table 4: KEGG pathway enrichment analysis by STRING (FDR < 0.05). Supplementary Table 5: lipids quantified in all ASC-CM samples, normalized on donor cell number, and ranked by coefficient of variation (CV). Supplementary Table 6: lipids quantified in all ASC pellets, normalized on donor cell number, and ranked by coefficient of variation (CV). [file 3086122.f1.zip › 3086122.f4.pdf]

Supplementary table 4_KEGG pathway enrichment analysis by STRING (FDR<0.05)

#term ID	term description	observed	backgrour	strength	false disco	matching	matching proteins in your network (labels)
hsa04060	Cytokine-cytokine receptor interaction	19	263	1.74	3.00e-28	9606.ENSF	TNFRSF1A,CSF3,CCL13,IL2,IFNG,IL4,IL5,CXCL13,CSF2,IL13,CSF1,IL17A,PDGFA,FAS,IL2RA,CXCL12,TNF,LTA,CCL4
hsa04630	Jak-STAT signaling pathway	9	160	1.63	2.28e-11	9606.ENSF	CSF3,IL2,IFNG,IL4,IL5,CSF2,IL13,PDGFA,IL2RA
hsa04657	IL-17 signaling pathway	8	92	1.82	2.28e-11	9606.ENSF	CSF3,IFNG,IL4,IL5,CSF2,IL13,IL17A,TNF
hsa05321	Inflammatory bowel disease (IBD)	7	62	1.93	6.97e-11	9606.ENSF	IL2,IFNG,IL4,IL5,IL13,IL17A,TNF
hsa05330	Allograft rejection	6	35	2.11	2.33e-10	9606.ENSF	IL2,IFNG,IL4,IL5,FAS,TNF
hsa04640	Hematopoietic cell lineage	7	94	1.75	7.21e-10	9606.ENSF	CSF3,IL4,IL5,CSF2,CSF1,IL2RA,TNF
hsa05323	Rheumatoid arthritis	6	84	1.73	2.21e-08	9606.ENSF	IFNG,CSF2,CSF1,IL17A,CXCL12,TNF
hsa04658	Th1 and Th2 cell differentiation	6	88	1.71	2.52e-08	9606.ENSF	IL2,IFNG,IL4,IL5,IL13,IL2RA
hsa04064	NF-kappa B signaling pathway	6	93	1.69	3.06e-08	9606.ENSF	TNFRSF1A,CCL13,CXCL12,TNF,LTA,CCL4
hsa04940	Type I diabetes mellitus	5	40	1.97	3.06e-08	9606.ENSF	IL2,IFNG,FAS,TNF,LTA
hsa04660	T cell receptor signaling pathway	6	99	1.66	3.58e-08	9606.ENSF	IL2,IFNG,IL4,IL5,CSF2,TNF
hsa04668	TNF signaling pathway	6	108	1.62	5.40e-08	9606.ENSF	TNFRSF1A,CSF2,CSF1,FAS,TNF,LTA
hsa05200	Pathways in cancer	9	515	1.12	9.54e-08	9606.ENSF	IL2,IFNG,IL4,IL5,IL13,PDGFA,FAS,IL2RA,CXCL12
hsa05162	Measles	6	133	1.53	1.52e-07	9606.ENSF	IL2,IFNG,IL4,IL13,FAS,IL2RA
hsa05166	HTLV-I infection	7	250	1.32	1.99e-07	9606.ENSF	TNFRSF1A,IL2,CSF2,PDGFA,IL2RA,TNF,LTA
hsa04664	Fc epsilon RI signaling pathway	5	67	1.75	2.05e-07	9606.ENSF	IL4,IL5,CSF2,IL13,TNF
hsa05310	Asthma	4	28	2.03	4.57e-07	9606.ENSF	IL4,IL5,IL13,TNF
hsa05332	Graft-versus-host disease	4	36	1.92	1.09e-06	9606.ENSF	IL2,IFNG,FAS,TNF
hsa04659	Th17 cell differentiation	5	102	1.57	1.21e-06	9606.ENSF	IL2,IFNG,IL4,IL17A,IL2RA
hsa05142	Chagas disease (American trypanosomiasis)	5	101	1.57	1.21e-06	9606.ENSF	TNFRSF1A,IL2,IFNG,FAS,TNF
hsa04672	Intestinal immune network for IgA production	4	44	1.84	1.97e-06	9606.ENSF	IL2,IL4,IL5,CXCL12
hsa05320	Autoimmune thyroid disease	4	49	1.79	2.82e-06	9606.ENSF	IL2,IL4,IL5,FAS
hsa05168	Herpes simplex infection	5	181	1.32	1.62e-05	9606.ENSF	TNFRSF1A,IFNG,FAS,TNF,LTA
hsa04151	PI3K-Akt signaling pathway	6	348	1.11	2.15e-05	9606.ENSF	CSF3,IL2,IL4,CSF1,PDGFA,IL2RA
hsa05143	African trypanosomiasis	3	34	1.82	5.69e-05	9606.ENSF	IFNG,FAS,TNF
hsa04380	Osteoclast differentiation	4	124	1.39	8.14e-05	9606.ENSF	TNFRSF1A,IFNG,CSF1,TNF
hsa04650	Natural killer cell mediated cytotoxicity	4	124	1.39	8.14e-05	9606.ENSF	IFNG,CSF2,FAS,TNF
hsa05418	Fluid shear stress and atherosclerosis	4	133	1.35	9.87e-05	9606.ENSF	TNFRSF1A,IFNG,PDGFA,TNF
hsa04010	MAPK signaling pathway	5	293	1.11	0.00012	9606.ENSF	TNFRSF1A,CSF1,PDGFA,FAS,TNF
hsa05144	Malaria	3	47	1.68	0.00012	9606.ENSF	CSF3,IFNG,TNF
hsa04217	Necroptosis	4	155	1.29	0.00016	9606.ENSF	TNFRSF1A,IFNG,FAS,TNF
hsa05164	Influenza A	4	168	1.25	0.00021	9606.ENSF	TNFRSF1A,IFNG,FAS,TNF
hsa04062	Chemokine signaling pathway	4	181	1.22	0.00027	9606.ENSF	CCL13,CXCL13,CXCL12,CCL4
hsa05140	Leishmaniasis	3	70	1.51	0.00032	9606.ENSF	IFNG,IL4,TNF
hsa05132	Salmonella infection	3	84	1.43	0.00053	9606.ENSF	IFNG,CSF2,CCL4
hsa05146	Amoebiasis	3	94	1.38	0.00071	9606.ENSF	IFNG,CSF2,TNF
hsa05145	Toxoplasmosis	3	109	1.32	0.0011	9606.ENSF	TNFRSF1A,IFNG,TNF
hsa04210	Apoptosis	3	135	1.22	0.0019	9606.ENSF	TNFRSF1A,FAS,TNF
hsa04932	Non-alcoholic fatty liver disease (NAFLD)	3	149	1.18	0.0024	9606.ENSF	TNFRSF1A,FAS,TNF
hsa05010	Alzheimer's disease	3	168	1.13	0.0033	9606.ENSF	TNFRSF1A,FAS,TNF
hsa05152	Tuberculosis	3	172	1.12	0.0035	9606.ENSF	TNFRSF1A,IFNG,TNF
hsa05167	Kaposi's sarcoma-associated herpesvirus infection	3	183	1.09	0.0040	9606.ENSF	TNFRSF1A,CSF2,FAS
hsa05014	Amyotrophic lateral sclerosis (ALS)	2	50	1.48	0.0046	9606.ENSF	TNFRSF1A,TNF
hsa05205	Proteoglycans in cancer	3	195	1.06	0.0046	9606.ENSF	PLAUR,FAS,TNF
hsa04612	Antigen processing and presentation	2	66	1.36	0.0074	9606.ENSF	IFNG,TNF
hsa04920	Adipocytokine signaling pathway	2	69	1.34	0.0079	9606.ENSF	TNFRSF1A,TNF
hsa04350	TGF-beta signaling pathway	2	83	1.26	0.0109	9606.ENSF	IFNG,TNF
hsa05322	Systemic lupus erythematosus	2	94	1.2	0.0136	9606.ENSF	IFNG,TNF
hsa04620	Toll-like receptor signaling pathway	2	102	1.17	0.0155	9606.ENSF	TNF,CCL4
hsa05165	Human papillomavirus infection	3	317	0.85	0.0155	9606.ENSF	TNFRSF1A,FAS,TNF
hsa04931	Insulin resistance	2	107	1.15	0.0163	9606.ENSF	TNFRSF1A,TNF
hsa04071	Sphingolipid signaling pathway	2	116	1.11	0.0186	9606.ENSF	TNFRSF1A,TNF
hsa05160	Hepatitis C	2	131	1.06	0.0229	9606.ENSF	TNFRSF1A,TNF
hsa05161	Hepatitis B	2	142	1.03	0.0262	9606.ENSF	FAS,TNF
hsa04150	mTOR signaling pathway	2	148	1.01	0.0278	9606.ENSF	TNFRSF1A,TNF
hsa05202	Transcriptional misregulation in cancer	2	169	0.95	0.0349	9606.ENSF	CSF2,PDGFA
hsa04015	Rap1 signaling pathway	2	203	0.87	0.0479	9606.ENSF	CSF1,PDGFA
