# Supplementary material for: Towards Secretome Standardization: Identifying Key Ingredients of MSC-Derived Therapeutic Cocktail
Source: Stem Cells Int. 2021 Aug 26;2021:3086122. doi: 10.1155/2021/3086122 (PMC8413055; doi:10.1155/2021/3086122)
Supplement: Supplementary Materials — Supplementary Table 1: protein concentration (pg/ml) of factors quantified in all ASC-CM samples. Supplementary Table 2: list of factors undetectable in all ASC-CM samples. Supplementary Table 3: factors quantified in all ASC-CM samples, normalized on donor cell number, and ranked by coefficient of variation (CV). Supplementary Table 4: KEGG pathway enrichment analysis by STRING (FDR < 0.05). Supplementary Table 5: lipids quantified in all ASC-CM samples, normalized on donor cell number, and ranked by coefficient of variation (CV). Supplementary Table 6: lipids quantified in all ASC pellets, normalized on donor cell number, and ranked by coefficient of variation (CV). [file 3086122.f1.zip › 3086122.f5.pdf]

Supplementary table 5_Lipids quantified in all ASC-CM samples, normalized on donor cell number and ranked by CV

	pg/10^6 ASCs		
Lipid ID	Mean	SD	CV (RSD)
SEA	78.8	19.7	25%
PGE2	27.1	7.7	28%
PEA	92.3	32.8	36%
PGF2	277.6	129.6	47%
DHA	1185.1	1111.2	94%
AA	20976.6	24167.6	115%
EPA	541.8	652.6	120%
