# Supplementary material for: Towards Secretome Standardization: Identifying Key Ingredients of MSC-Derived Therapeutic Cocktail
Source: Stem Cells Int. 2021 Aug 26;2021:3086122. doi: 10.1155/2021/3086122 (PMC8413055; doi:10.1155/2021/3086122)
Supplement: Supplementary Materials — Supplementary Table 1: protein concentration (pg/ml) of factors quantified in all ASC-CM samples. Supplementary Table 2: list of factors undetectable in all ASC-CM samples. Supplementary Table 3: factors quantified in all ASC-CM samples, normalized on donor cell number, and ranked by coefficient of variation (CV). Supplementary Table 4: KEGG pathway enrichment analysis by STRING (FDR < 0.05). Supplementary Table 5: lipids quantified in all ASC-CM samples, normalized on donor cell number, and ranked by coefficient of variation (CV). Supplementary Table 6: lipids quantified in all ASC pellets, normalized on donor cell number, and ranked by coefficient of variation (CV). [file 3086122.f1.zip › 3086122.f6.pdf]

Supplementary table 6_Lipids quantified in all ASC pellets, normalized on donor cell number and ranked by CV

	pg/10^6 ASCs		
Lipid ID	Mean	SD	CV (RSD)
DHA	51177.2	5271.8	10%
AA	1052849.3	110864.3	11%
EPA	96793.3	19380.6	20%
PGE2	1230.6	246.4	20%
AEA	72.0	16.6	23%
SEA	1472.0	448.5	30%
PGF2	423.5	131.4	31%
PEA	1198.7	547.1	46%
2AG	1079.3	518.6	48%
OEA	920.6	463.1	50%
LEA	198.1	110.1	56%
